# Unintended pregnancy: a framework for prevention and options for midlife women in the US

**DOI:** 10.1186/s40695-017-0027-5

**Published:** 2017-09-15

**Authors:** Versie Johnson-Mallard, Elizabeth A. Kostas-Polston, Nancy Fugate Woods, Katherine E. Simmonds, Ivy M. Alexander, Diana Taylor

**Affiliations:** 1 Department of Family, Community, and Health System Science, Robert Wood Johnson Nurse Faculty Scholar Alum, University of Florida, College of Nursing, Gainesville, FL USA; 2 Daniel K. Inouye Graduate School of Nursing, Uniformed Services University of the Health Sciences, Bethesda, MD USA; 3 Biobehavioral Nursing and Health Informatics, Interim Associate Dean for Diversity, Equity, and Inclusion, University of Washington School of Nursing, Seattle, WA USA; 4 MGH Institute of Health Professions, Boston, MA USA; 5 Director of Advance Practice Programs, Storrs, CT USA; 6 UCSF School of Nursing, Research Faculty, Advancing New Standards in Reproductive Health Program (ANSIRH), UCSF Bixby Center for Global Reproductive Health, University of California, San Francisco, CA USA

**Keywords:** Reproductive health, Menopause, Contraception, Pregnancy, Abortion, Transition, Framework, Age, Women, Sex

## Abstract

Recently unintended pregnancies have been described as "a new kind of mid-life crisis." Given the high prevalence of unwanted or mistimed pregnancy in the US, we examined the sexual and reproductive health patterns of sexually active midlife women. An examination of the prevalence of unintended pregnancy among midlife women revealed a gap in data indicating unmet sexual and reproductive health needs of midlife women. The application of a framework for primary, secondary and tertiary prevention for unintended pregnancy may assist with guiding care for women and identifying implications for reproductive health policy and potential political interference as they relate to sexual and reproductive health in midlife women.

## Background

An unintended pregnancy is one that was mistimed or unwanted. Mistimed pregnancies are those that occur among women who do not want to become pregnant at the time the pregnancy occurred, but who want to become pregnant at some time in the future. Unwanted pregnancies are those that women experience when they do not want to become pregnant then or at any time in the future. An intended pregnancy is one that is desired at the time it occurred or sooner [1]. Although one might imagine that adult women, in particular midlife women, would be experienced in fertility control and family planning, even older women do not seem to be immune to the experience of unintended pregnancy [2].

James [3] conducted a systematic review of studies of multiple unintended pregnancies spanning the period from 1979 to the present. She found 8 studies that provided incidence rates on multiple unintended pregnancies ranging from 7.4 to 30.9 per 100 person-years and prevalence rates ranging from 17% to 31.6%. In addition, she examined factors associated with multiple unintended pregnancies: increasing age, identifying as Black or Hispanic, having an income below the poverty level, experiencing a non-voluntary first sexual intercourse and especially at a very young age, participating in sex trade, experiencing stressful life events, and having had a previous abortion. Factors associated with reduced risk of multiple unintended pregnancies were use of IUDs or combined oral contraceptives. Some of these risk factors are modifiable, for example, contraceptive type and use. Others reflect pervasive effects of poverty and other social determinants of health disparities.

In addition to the above reasons for unintended pregnancy among midlife women, sexual health of midlife women is often over-looked by both primary care providers and researchers, with most effort focused on younger women. Nonetheless, midlife women are at greater risk of new sexually transmitted infections and unintended pregnancy than previously imagined [4]. In addition to recent changes in relationships that make many women single again, a limited knowledge of safer sexual practices, less predictable menstrual cycles, and health care providers who may not evaluate sexual health risks among this population of women may contribute to the incidence of unintended pregnancy [4].

The U. S. has the highest rates of unintended pregnancy among the most developed nations of the world, with nearly half of pregnancies being unintended [1]. Despite efforts to improve access to evidence-based and culturally sensitive reproductive health care, outcomes of unintended pregnancy prevention efforts in the United States lag behind those in many other countries. Some progress in reducing the risk of unintended pregnancy can be attributed to the implementation of the Affordable Care Act (ACA) from 2009 to 2016 [5]. The ACA improved coverage for contraceptive services for adolescents and young adult reproductive age women up to 26 years of age. In addition, provisions required insurance plans to cover contraceptives for all women, regardless of their age, with no out of pocket cost required [5].

Given the high prevalence of unintended pregnancy in the US, the purposes of this paper are to:

- describe sexual and reproductive health (SRH) patterns of sexually active midlife women;
- examine the prevalence of unintended pregnancy among midlife women;
- apply a framework for primary, secondary and tertiary prevention for unintended pregnancy grounded in a primary health care perspective to midlife women; and
- explore reproductive health policy and potential political interference as they relate to sexual and reproductive health in midlife women.
  Sexual and Reproductive Health Patterns of Midlife Women.

### Why is sexual and reproductive health important for midlife women?

Women remain sexually active well into their postmenopausal years and many sexually active midlife women not using an effective family planning method are at risk for pregnancy. Evidence supports pregnancy in sexually active women can occur over the age of 50 years, with women remaining potentially fertile. Sexually active couples are at risk for pregnancy until women reach approximately 54 years, at which age menopause has occurred in 95% [6]. Indeed, women are often advised to continue using birth control/family planning methods until they have not had a menstrual period for one calendar year.

The menopausal transition challenges women to manage their fertility and complicates their family planning efforts. As they begin the menopausal transition, women’s menstrual cycles become irregular, with some experiencing long periods (months) of amenorrhea during the latter part of the menopausal transition prior to their final menstrual period [7]. In general, this period of amenorrhea of variable duration has stimulated guidelines that one year of amenorrhea be observed prior to confirming a women’s cycle pattern as consistent with post-menopause. Despite this conservative definition, there are rare occurrences of another menses following a year of amenorrhea.

Mercer and colleagues surveyed midlife women in the United Kingdom, finding that those 35–44 years reported an average of 4 episodes of sexual intercourse over the past month and those 45–54 reported 3.5 episodes [8]. Similar data from women in the U.S. by Finer and Philibin reported 75% of women aged 40 to 44 years were sexually active, a small percentage 4% of the 75% reported not using contraception and actively trying to conceive [9].

In Great Britain, 25% of women 40–44 years of age reporting using no contraception, compared with 28% of women 45–49 years of age [8]. In a similar study conducted with a cohort of sexually active women 40–44 years in the USA, 31% of women reported not using any form of contraception [10]. As seen in the data in Table 1, use of hormonal contraceptives was low in both the UK and the US. In both countries, women 40–44 years of age relied on female and partner sterilization and male condoms, with the latter being used by a greater proportion of those in the UK [10]. In the US 13% reported relying on vasectomy/partner sterilization, 35% relied on female sterilization compared to 25% of UK participants relying on partner sterilization and 15% on female sterilization [8, 11]. Thus US and UK data indicate a majority of midlife women are using condoms, male and female sterilization [8–10]. The U. S. data on contraceptive use collected by National Survey of Family Growth (NSFG) included women aged 15–44 years. These data did not provide information about the latter midlife years. Only recently (2015) has data collection expanded to include upper bound of age range 44 to 49 years.

**Table 1:** Contraceptive Method of Women 40–44 years in Great Britain and U.S

Age 40–44 years	Great Britain %	United States
Male condom	21%	8%
Pill	13%	8%
IUD	8%	<1%
Hormonal IUS	7%	<1%
Injection	3%	1%
Implant	1%	<1%
Patch	<1%	1%
Female Sterilization	15%	35%
Partner Sterilization	25%	13%

Adapter from: Finer, L. B., & Philbin, J. M. (2014). Trends in ages at key reproductive transitions in the United States, 1951–2010. Women’s Health Issues, 24(3), e271-e279; Hardman, S. M., & Gebbie, A. E. (2014). The contraception needs of the perimenopausal woman. *Best Practice & Research Clinical Obstetrics & Gynaecology*, *28*(6), 903–915

One might also ask whether women are confused about using hormone therapy (HT) to manage symptoms related to menopause and hormones used in contraceptive methods. Although hormone therapy does not provide effective contraceptive effects, both hormone therapy and hormonal contraceptives can mask the onset of menopause by stimulating regular withdrawal bleeding. Use of hormonal contraception and hormone therapy (HT) for menopause-related symptoms in combination is not recommended. Once ovulation inhibition is no longer a concern, lower dosed menopause-specific methods can be considered for managing symptoms such as hot flashes that are not well-controlled by other means. Risks for adverse clinical outcomes exist for women continuing hormonal contraceptive use after menopause [11].

## Prevention of unintended pregnancy

### What are the risks of unintended pregnancy in midlife women?

Data on unintended pregnancy rates in U.S. among women older than 45 years does not appear to be intentionally collected by the NSFG at the time of writing this paper. Estimates are that for women 40–44 years of age, 48% of pregnancies are unintended [12–15]. However, Europe has reported unplanned pregnancy estimates as high as 30% among women 45–49 years of age [12, 13].

In the United States, birth rates for women up to age 44 years have been trending upward since the 1990s with 0.3 births per 1000 to 0.7 births per 1000 in 2012 to 0.8 births /1000 in 2013 [15, 16]. The increase in live births among some midlife women in the U.S. is reportedly due to planned births and increasing use of assisted reproductive technology [17]. Unintended pregnancy rates for nearly half of U.S. women 40–44 years old and the international data from Europe reporting unplanned pregnancy rates as high as 30% in women 45–49 years of age are concerning. Framing and addressing unintended pregnancy at a global level is imperative for the health of women and children.

## Prevention and Management of Unintended Pregnancy for midlife women: A framework

### What is a framework for prevention and management of unintended pregnancy in midlife women?

Taylor and colleagues proposed using a comprehensive, culturally appropriate public health framework in which primary, secondary, and tertiary measures are integrated into nationally supported clinical guidelines and incorporated into primary care competencies for health professionals [13]. The proposed framework for prevention and management of unintended pregnancy rests on foundational work by the World Health Organization on Primary Health Care that is grounded in public health and primary medical care. Such a framework is used widely in national health services outside the U.S., e.g. Canada, UK. In this framework, public health care models include primary, secondary, and tertiary prevention strategies. (In the US primary, secondary, and tertiary care refers to both settings and type of clinical care and is not systematically linked to a public health framework).

In the public health model proposed in this paper, primary prevention includes services designed to promote intended, healthy pregnancies with healthy mothers and infants and reduction of personal perinatal, neonatal, and family adverse events. Primary prevention services incorporate preconception care, reproductive life plan development and evaluation and contraception and emergency contraception dispensing or prescribing. Secondary prevention services are focused on identification of unintended pregnancies early in order to improve reproductive health outcomes. Secondary prevention services incorporate pregnancy diagnosis, pregnancy options counseling and management, referral and counseling for pregnancy care, adoption or early abortion referral and care. Tertiary prevention is focused on preventing complications associated with a later unintended pregnancy and support for women and their families who experience later unintended pregnancy [13]. Tertiary prevention for midlife women may incorporate prevention efforts specific to women who have experienced multiple unintended pregnancies [13].

## Primary prevention of unintended pregnancy

### Why primary prevention of unintended pregnancy?

The concept of preconception health is important for girls and women from birth to death, with the goal being optimal health which may involve getting healthy, staying healthy, and managing health problems. Preconception health is not age-specific but based on individual health, and not only for those planning a pregnancy, since about half of pregnancies are unplanned. The concept of preconception health is important for midlife women. Embedded within the concept of preconception health are the notions of the *Well Woman Visit,* an opportunity to promote health by addressing health concerns and educating women about pregnancy risks. Women experience aging differently and their care should be individualized. Aging into midlife should not be a barrier to addressing sexual health topics. Midlife women have sexual concerns and sometimes may want their healthcare provider to breach the subject first, thereby opening the door to two way and trusting conversation.

Nurses, physicians and other providers of primary prevention services for midlife women during transition to menopause should incorporate counseling and education in regard to fluctuating fertility and methods to prevent unplanned pregnancies. The incorporation of reproductive life plan development and topics of discussion should include safe and effective contraceptive methods, diagnosis and management of common age-related medical conditions such as hypertension, diabetes and breast cancer, transition to menopause and hormone therapy to manage menopause-related symptoms. The American College of Obstetricians and Gynecologists guidelines recommend family planning counseling and contraceptive protection for women at risk for pregnancy until they are 55 years old [14]. Safe and effective contraceptive methods for midlife women exist in many forms and delivery methods (see Table 2).

**Table 2:**
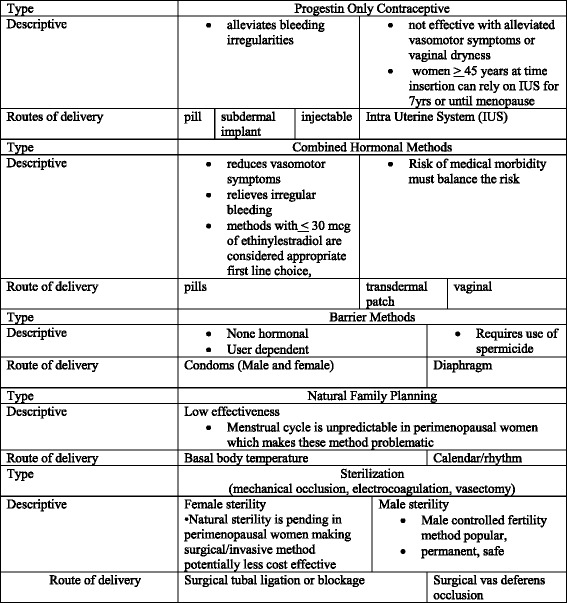
Contraceptives methods: Type, details and route of delivery

Adapted from Finer, L. B., & Philbin, J. M. (2014). Trends in ages at key reproductive transitions in the United States, 1951–2010. Women’s Health Issues, 24(3), e271-e279

10. Hardman, S. M., & Gebbie, A. E. (2014). The contraception needs of the perimenopausal woman. Best Practice & Research Clinical Obstetrics & Gynaecology, 28(6), 903–915

Midlife women at risk for pregnancy may have a twofold benefit from use of certain contraceptive methods. Hormonal contraceptives can help alleviate irregular menstrual bleeding, hot flashes, night sweats and vaginal dryness while simultaneously lowering pregnancy risk. Contraceptive methods should be individualized based on the women’s health history: for example, contraceptives containing estrogen may not be ideal for women with hypertension, diabetes, cancer or other chronic medical conditions. Newer combined oral contraceptive (COC) preparations contain low dose estrogen and progestogen and are safe, and monophasic pill with 30 mcg or less of estrogen are considered an appropriate first line choice, safe and effective for women with chronic medical conditions [10, 11, 18]. (see Table 2). Women using contraceptive methods can be advised to stop contraception at age 55 providing they have stopped menses for at least one year.

### What does pregnancy risk look like at midlife?

Godfrey et al.^,^ estimated that over three-quarters of women aged 45–50 were at risk for an unintended pregnancy due to their low use of contraception [14]. Many midlife women believe they are no longer able to conceive and may choose not to use contraception. For others, the use of hormonal contraception becomes riskier if the woman has a medical conditions that increase the risks associated with a particular method (e.g., combined estrogen-progestogen contraceptives) [15]. Healthcare providers, too, fail to educate midlife women about important non-contraceptive benefits of hormonal contraception, including reduction of vasomotor symptoms, treatment of abnormal uterine bleeding, decreased risk of ovarian and endometrial cancer, and maintenance of bone mineral density. For example, the levonorgestrel (LNG) intrauterine system (IUS) (a long-acting reversible contraceptive method), is a first-line therapy for heavy menstrual bleeding which is commonly experienced during midlife [15]. Perhaps more importantly, the LNG-IUS is not only an alternative to uterine ablation and hysterectomy, but also provides superior contraceptive effectiveness (equivalent to sterilization) [16].

As fertility declines in midlife, recognition of pregnancy may become more problematic. Changes in a midlife woman’s body, such as changing bleeding patterns or prolonged periods of amenorrhea, may be mistaken as normal, instead of as signs of an unintended pregnancy [17]. Vasomotor symptoms (e.g., hot flashes), changes in menstrual patterns, body weight, vaginal discharge, and mood may be interpreted by the midlife woman as indicating menopause [17]. With advancing age, midlife women with chronic gynecological issues (e.g., uterine fibroids, endometriosis) also experience decreased fertility. It would be reasonable, then, for a midlife woman not to think about the risk of unintended pregnancy when her understanding of her changing body is related to “*the change of life”* or to her gynecological issue.

## Secondary prevention of unintended pregnancy

Secondary prevention of unintended pregnancy optimizes early diagnosis of a pregnancy. Clinical care for women who suspect unintended pregnancy begins with a health history and physical exam, as well as engagement with women to support their decision process.

*Why a health history?* Obtaining a health history is the first step during a pregnancy testing encounter. Open-ended questions are used to gather information about a midlife woman’s physical, psychological, social, and sexual history. Because it is common for midlife women to experience unpredictable menstrual bleeding, such as skipping a month or having multiple bleeding episodes in a month, they may experience difficulty with estimating gestational age. Therefore, a woman’s sexual and other health history will serve as a database on which to date gestational age, as well as to provide insight as to how a woman feels about the possibility of a pregnancy or termination.

### Why a physical examination?

In addition to obtaining a health history, a physical examination may be helpful when attempting to diagnose a pregnancy. If too early in a pregnancy, physical changes of the vagina, cervix, and uterus may not be evident. Inconsistencies among and between a woman’s health history, physical examination, and pregnancy test results may warrant a quantitative (serum) pregnancy test and/or transvaginal ultrasound.

### What is emergency contraception?

If desired, within 5 days of unprotected sexual intercourse, a midlife woman should be offered emergency contraception (EC). EC consists of methods that can be used to prevent pregnancy. Effectiveness of EC methods vary depending on the method and most importantly, timing of administration. In the United States (US), four options are available (the Cu-IUD [intrauterine copper contraceptive] and three types of emergency contraceptive pills [ECPs]). See Table 3, for EC types, timing of initiation, and evidence summary. It is critically important to note that not all midlife women are eligible to use all forms of EC. Healthcare providers should refer to the *U.S. Medical Eligibility Criteria for Contraceptive Use, 2016* [18] for guidance as to whether women with particular medical conditions or lifestyle behaviors can use specific EC methods.

**Table 3:** Types of Emergency Contraception

Intrauterine Device (IUD)		
IUD	Initiation of EC	Evidence Summary
Cu-IUD (intrauterine copper contraceptive)	• Can be inserted within 5 days of 1st act of unprotected sexual intercourse as an EC. • Additionally, when can estimate the day of ovulation, can be inserted beyond 5 days after sexual intercourse (as long as insertion does not occur >5 days after ovulation).	• Highly effective. • Can be continued as regular contraception (Cleland et al., 2012).
Emergency Contraceptive Pills (ECPs)		
ECPs	Initiation of EC	Evidence Summary
Ulipristal acetate (UPA) • Single dose (30 mg)	• Take as soon as possible within 5 days of unprotected sexual intercourse.	• Similar effectiveness to Cu-IUD when taken within 3 days after unprotected sexual intercourse. • Shown to be more effective than LNG formulation 3–5 days after unprotected sexual intercourse (Glasier et al., 2010).
Levonorgestrel (LNG) • Single dose (1.5 mg) or • Split dose (1 dose of 0.75 mg of levonorgestrel, followed by a 2nd dose of 0.75 mg of levonorgestrel 12 h later	• Take as soon as possible within 5 days of unprotected sexual intercourse.	• Similar effectiveness to Cu-IUD when taken within 3 days after unprotected sexual intercourse (Glasier et al., 2010). • LNG may be less effective than UPA in obese women (Jatlaoui, 2016).
Combined estrogen and progestin in 2 doses (Yuzpe regimen) • 1 dose of 100 *μ*g of ethinyl estradiol plus 0.50 mg of levonorgestrel followed, by a 2nd dose of 100 μg of ethinyl estradiol plus0.50 mg of levonorgestrel 12 h later	• Take as soon as possible within 5 days of unprotected sexual intercourse.	• Less effective than UPA or LNG. • Associated with more frequent occurrence of side effects (nausea and vomiting) (Raymond et al., 2004).

Adapted from Curtis et al. (2016). U.S. Selected Practice Recommendations for Contraceptive Use, 2016. MMWR Recomm Rep 2016;65(No. RR-4): [1–66]

### Why pregnancy confirmation?

A woman may suspect pregnancy and seek confirmation, or be unaware of the possibility of pregnancy due to increasingly sporadic menstrual periods during midlife. She may choose to either perform an over-the-counter pregnancy test in the privacy of her home, or visit her healthcare provider to appraise the cause of her amenorrhea. During visit with the healthcare provider, a pregnancy test and ultrasound may be performed. The pregnancy test results and ultra sound gestational age assessment are shared with the woman. Simmonds and Likis [19] have identified four steps for delivering options counseling after a confirmed pregnancy: 1) exploring feelings about the pregnancy, 2) identifying support systems and assessing risks, 3) assisting with decision making, and 4) providing desired service or referral.

### How to explore feelings about the pregnancy?

When exploring a woman’s feelings, healthcare providers should use open-ended questions which will allow the woman to freely express her thoughts or concerns. A starting point may include, “How do you feel about this pregnancy?” Such a question will create a safe space for the woman, as well as allow the healthcare provider to appraise whether she has made any decisions about the pregnancy and provide insight into her understanding of available options (Table 4).

**Table 4:** Open-ended Questions to Facilitate Communication When Exploring Pregnancy Options

Exploring Feelings
• “How do you feel about this pregnancy?” • “I want to be sure that you know what all of your options are, and I will help you get good care no matter what you decide to do about this pregnancy.” • “Tell me what you have heard about adoption?” • “Tell me what you have heard about abortion?” • “Do you have any questions about what it would be like to be a parent?” • “Do you have any questions about what it would be like to place a child for adoption?” • “Do you have any questions about what it would be like to have an abortion?”

*Adapted from Simmonds, K. & Likis, F. E. (2011).* Caring for women with unintended pregnancies. *Journal of Obstetric, Gynecologic, and Neonatal Nursing, 40(6), pp. 794–807; and Simmonds, K. & Stern, L. (2017). The challenge of unintended pregnancies. In Alexander, I., Johnson-Mallard, V., Kostas-Polston, E. A., Fogel, C. I., & Woods, N. F. (Eds.), Women’s Health Care in Advanced Practice Nursing. New York: Springer Publishing Company, LLC*

### Why identify support systems and assess risks?

Identifying support systems and social risk of violence, abuse, and/or sexual and reproductive coercion are important for the physical and mental well-being of a woman. Pregnancy resulting from non-consensual sexual intercourse may require involving a Social Worker, Psychologist, and/or Psychiatrist in the care of the pregnant woman. What is more, such situations necessitate careful contemplation and collaboration, on behalf of the pregnant woman, as mandated reporting may further compromise a woman’s health and well-being.

There appears to be no link between abortion and adverse mental health outcomes like depression [20, 21]. However women with pre-existing mental health problems and those with a history of sexual abuse and/or intimate partner violence are at risk for mental health issues. Robinson, Stotland, and Russo determined the best predictor of serious mental health issues after abortion was emotional health prior to abortion [22]. Obtaining an accurate and thorough woman’s physical, psychological, social, and sexual health history will help the healthcare provider identify and treat pre-existing psychiatric problems and psychosocial stressors.

### Why assist with decision-making?

Prior to presenting for a healthcare encounter a woman who suspects pregnancy may have already made her decision about the pregnancy. She may have decided to continue with the pregnancy and keep the baby, to continue with the pregnancy and relinquish the baby for adoption, or to have an abortion. Once a decision has been made a referral for appropriate services can help provide a seamless transition.

### Are coordination of services and referral important for midlife women?

Providing or referring for appreciate services is essential for transition of care for the midlife woman experiencing an unintended pregnancy. Helping her to make appointments and navigate the system is an essential component of reproductive health care services, particularly when women must seek pregnancy termination outside of the care system, county, or state. Should she decide to continue the pregnancy, a woman should establish obstetrical care and begin prenatal care. If she decides to relinquish the baby for adoption a referral to an adoption agency will be needed. If she decides to terminate the pregnancy, referral for safe, competent abortion care is also essential transition care. Although referral patterns are often clear for other health problems midlife women encounters, these arrangements are often left to the women to navigate alone.

### Why pregnancy options counseling?

Regardless of whether intended, unwanted, mistimed, or ambivalent, pregnancy is a life-changing event. Providing counseling and information regarding pregnancy options will be guided by a woman’s circumstance and desires. Regardless of circumstance, the possibility of an unintended pregnancy becomes a woman’s private health matter—oftentimes, a burden for her to bear. Each woman’s situation, presentation, and timing for appraisal of pregnancy exposure or pregnancy confirmation, lend themselves to individual but related healthcare encounters: pregnancy confirmation and counseling on options (i.e. maintaining the pregnancy, relinquishing the baby for adoption, or abortion). A woman should be allowed to self-identify what she perceives are viable options. Parenting, adoption, and abortion are options. Many factors may influence women’s decision making. It is important that healthcare providers establish a climate of trust and deliver non-judgmental active listening.

### Is maintaining pregnancy an option?

Recent studies suggest that women who carry unwanted pregnancies to term are likely to be in poverty, have depressive symptoms, and have reason to worry about the negative impact of an unintended birth on that child as well as on their existing children [20, 23, 24].

Choosing to continue with an unwanted pregnancy can be emotionally painful and negatively impact a woman’s mental health. Herd, Higgins, Sicinski, and Merkurieva^25^ examined the association between unwanted and mistimed pregnancies and mental health in women whose pregnancies occurred prior to the legalizaton of abortion [25, 26]. Experiencing unwanted pregnancies, especially after a woman or couple has reached a desired number of children, appears to be strongly associated with poor mental health effects for women later in life. Although not statistically significant, the authors reported more depressive symptoms and a greater likelihood of having a significant episode of depression in the now midlife women who carried an unwanted pregnancy to term and raised the infant. Caretaking stressors; social and economic burdens; changes in educational, career, and health trajectories; and poorer quality relationships between the parent/s and child were posited as causative.

A study to assess differences in child health and development outcomes for women who were denied an abortion and carried an unwanted pregnancy to term compared with women who obtained an abortion found lower scores on child development for the children of women denied abortions [20, 24]. Evidence suggests that unintended births may lead to poorer quality relationships between parents and children thereby negatively influencing parental well-being [21]. Therefore, when midlife women seek out a healthcare provider, the provider has a window of opportunity for detection and intervention. In such cases, a multidisciplinary approach to prenatal care will be in the best interest of both the pregnant woman and her future child.

### Is adoption an option?

It is estimated that approximately 14,000 women choose adoption annually [27, 28]. Most unintended pregnancies result in a woman choosing to keep the infant or have an abortion. In fact, only 3% of US White unmarried women and fewer than 2% of Black unmarried women are estimated to place an infant for adoption during their reproductive lifetime [27, 28]. Data regarding the number of infants relinquished each year, and the demographics of women who relinquish their parental rights to infants (birth mothers) is limited; largely due to the low number of infants put up for adoption (Child Welfare Information Gateway, 2011) [27]. The small proportion of women who choose relinquishment of parental rights makes it difficult to collect and generalize data concerning this population [27]. In the US, federal legislation ensures that all pregnant women are offered the opportunity to receive impartial information regarding prenatal care and delivery; infant care, foster care, or adoption; and pregnancy termination (Title X of Public Law 91–572, Section 1008, 1970) [29].

Further, adoption laws differ by state, therefore it is important that healthcare providers are able to: 1) counsel women and provide state-specific information regarding the different options of adoptions, including the different manners in which adoptions can be processed (e.g., public or private agency, adoption lawyer), 2) discuss with the woman, her rights as a birth mother, and 3) refer the woman to an impartial adoption professionals such as social worker [19, 30]. Women considering adoption should establish prenatal care as well as be provided information reinforcing prenatal care and education. The healthcare provider should also review hospital policies regarding adoption with the woman well in advance of her delivery [19, 30].

### Is abortion an option?

Women come to a decision to terminate a pregnancy for many different reasons: the most commonly reported include concerns or responsibilities to care for others, inability to afford a child, and the belief that having a baby would interfere with other work and life commitments [31]. For midlife women, concerns related to their age, such as the increased risk of a fetal genetic anomaly or the belief that their family is complete, may also be a factor in their decision. Currently, 19% of all pregnancies in the US end in abortion; most (90%) take place in early pregnancy (before 13 weeks’ gestation) [32], and are performed or initiated in ambulatory clinic [33, 34].

### What is abortion counseling?

Women who decide to have an abortion should be informed about the estimated gestational age of the pregnancy and encouraged to seek care as early as possible, as the risks of abortion increase in more advanced gestations, and it is also more expensive and can be harder to obtain. It is important that women understand that delayed decision making may be problematic as advanced gestational age abortions are associated with poorer maternal health outcomes, may be difficult to obtain, and/or may be illegal in some states. Recent evidence suggests that carrying an unintended pregnancy to term or being denied an abortion raises an important policy issues affecting women’s health. When women do not have freedom to make a decision or cannot act on their decision to end a pregnancy, they may experience long-term psychological sequelae. A recent study of women’s mental health and well-being five years after receiving or being denied an abortion (The Turn-away Study) revealed that women denied abortion reported more anxiety symptoms, lower self-esteem, lower life satisfaction, and similar levels of depression [24, 35]. Being denied an abortion [and continuing an unwanted pregnancy] was associated with greater risk of initial experience of adverse psychological outcomes. Over time, psychological well-being improved with both groups of women having similar levels of well-being [24].

Often the decision to have an abortion, as compared to other health decisions can be a subject of conflict. To explore this belief, researchers used the *Decisional Conflict Scale* (DCS), an instrument widely used in many health specialties and considered the gold standard for measuring decisional conflict, as well as the Taft-Baker Scale (TBS), a valid and reliable instrument for use to measure decisional certainty in women seeking abortion and to predict a decision to continue a pregnancy [23, 36]. The majority of women (ages 15 and older), reported that they were certain of their decision when presenting for abortion care. Similar to other studies, women reported that although the decision to have an abortion was not an easy one, they were confident that they had made the right decision [23, 36].

What are the current abortion demographics? Nationally, the abortion rate has fallen in recent years, from a historic high of 29.3/1000 women in 1980–81 to 14.6/1000 in 2014 [1, 33]. This trend has been attributed to the combined effects of increases in the use of highly effective contraceptive methods, as well as the widespread passage of restrictive abortion legislation that makes it more difficult for some women to access services [33]. Though the overall rate has declined, on closer examination, disparities in utilization and inequities in access to services are evident. Women who are poor and low-income, Black, and young are disproportionately represented among abortion patients [33]. Disproportionate patterns exist that mirror those previously discussed with unintended pregnancy (See Table 5), and which are inextricably linked to the broader contexts of unequal access to health care, economic resources and education, and other social determinants of health [37, 38].

**Table 5:** Demographics of Women Obtaining Abortions in 2014: Age, Race/Ethnicity, Relationship Status and Sexual Orientation

AGE	PERCENT	RACE/ETHNICITY	PERCENT
Younger than 20	11.9	White	38.7
20–29	60.0	Black	27.6
30–34	15.9	Hispanic	24.8
35–39	9.1	Asian/Pacific Islander	5.5
Older than 40	3.1	Other	3.4
RELATIONSHIP STATUS	PERCENT	SEXUAL ORIENTATION	PERCENT
Married	14.3	Heterosexual	94.4
Living together not married	31.0	Homosexual	0.3
Single/never married/not living together	45.9	Bisexual	4.2
History of being married, not living together	8.8	Something else	1.1

Adapted from Jones RK, Finer LB, Singh S. Characteristics of US abortion patients, 2008. New York: Guttmacher Institute, 2010, 20,101–8

With regard to age, in 2014 women age 40 and older had low rates of abortion (3.1/1000) compared to all other age groups [38]. While a table demonstrating the specific demographics among midlife women who experience unintended pregnancy and undergo abortion would likely be very illuminating, such data are not readily available [39]. Data on unintended pregnancies and abortion are published in the aggregate. More often specific demographics are available for younger women. Perhaps this dearth of data is a reflection of the common misperception that midlife women are not sexually active, and therefore may not be at risk for unintended pregnancy. Furthermore, midlife women, even those who are known to be sexually active, are infrequently counseled about contraception.

Between 2008 and 2014, however, this older group experienced a modest increase (6.2%) in abortion utilization, which contrasts with the dramatic declines (25.2–44%) observed among women between 15 and 20 years old during the same period. This decrease in rates among adolescents has been linked to broader declines in teenage pregnancy, which are not attributable to changes in patterns of sexual activity or contraceptive use, but rather are theorized to be a result of greater educational opportunities, and media and economic influences [38, 40].

### Are there different abortion methods and providers?

The Centers for Disease Control and Prevention (CDC) defines “legal induced abortion” as an intervention performed by a licensed clinician (e.g. a physician, nurse-midwife, nurse practitioner or physician assistant) that is “intended to terminate an ongoing pregnancy” [41]. In surveillance reporting, most states and systems distinguish between two major categories of abortion, “surgical” and “medical,” however the alternative terms “aspiration” and “medication” have been advanced, as the former “obfuscates the differences in the procedures and the training requirements for provision, as well as evokes scary imagery that contributes to wider misunderstanding” ([42], p78).

Regardless of the terminology, procedural abortion involves the removal of pregnancy and supporting endometrial tissue from the uterus via electric or manual vacuum aspiration [42]. In gestations of greater than 14–15 weeks, the procedure typically requires the use of additional instrumentation, a method referred to as “dilation and evacuation” (or D & E). Alternatively, abortion can be provoked by the administration of medication. A combination of the drugs mifepristone and misoprostol is the most common regimen used for early abortions in the US [42]. These medications interrupt pregnancy development and stimulate its expulsion from the uterus. Medication can also be administered later in pregnancy to stimulate uterine contractions that lead to the passage of a fetus. This approach – commonly referred to as “labor induction abortion” – is an uncommon method of abortion in the US at this time (<1.0%) [43].

Uterine aspiration is currently the most common method of abortion in the US, comprising approximately 77% of all abortions [43]. Due to outdated state laws, only physicians are legally permitted to perform this procedure in most states [43]. In abortions beyond 14–15 weeks’ gestation, abortion is restricted to physicians with advanced clinical training and skill. In 2016, the Food and Drug Administration (FDA) revised the label for mifepristone to expand the type of licensed healthcare providers, however, as of this writing, 37 states still require a physician to prescribe the medication, even though advanced practice nurses and physician assistants in many states have prescriptive authority. Though laws and regulations in the majority of states require a physician to provide or be involved in the delivery of abortion services, in most settings care is provided by multi-disciplinary teams that include a range of health care workers such as medical assistants, nurses, counselors, social workers, physician assistants, and others. See Table 6 for a comparative overview of medication and aspiration abortion, the two most common methods in the US.

**Table 6:** Early Abortion methods

	Medication abortion	Aspiration abortion
Efficacy	95–98%	99%
Gestational age eligibility	Can use up to 10 weeks’ gestation	Up to 14–15 weeks’ gestation
Typical number of visits to abortion provider	2 (one to initiate process; one to confirm completion of abortion)	Typically 1–2; one for procedure; follow up can be with abortion provider or primary care provider
Advantages	• Does not require invasive procedure • Some women feel it is more “natural” • Offers more privacy as abortion occurs at home (or other chosen place) • May be accessible in remote/less-densely populated areas	• Complete within a short, defined period of time (several minutes) • Trained health personnel are present throughout procedure • Bleeding is typically light after the procedure
Disadvantages	• Process can take hours to complete • Failure of method requires aspiration of uterus • Cramping can be strong, and last longer than with aspiration abortion • Heavy bleeding is common	• Requires instrumentation of uterus • Providers generally located in areas with higher density populations • Pain medication and anesthesia can cause side effects

Adapted from the Reproductive Health Access Project. (2016). Early Abortion Options, Retrieved from http://www.reproductiveaccess.org/wp-content/uploads/2014/12/early_abortion_options.pdf **;**

University of California at San Francisco Medical Center. (2016). Medical versus Surgical Abortion, Retrieved from https://www.ucsfhealth.org/education/medical_versus_surgical_abortion/

And the Center for Reproductive Health in Family Medicine. (n.d.) Comparison of early abortion options. Retrieved from http://www.earlypregnancylossresources.org/resources/clinical-resources/

### Does disparity in abortion access exist?

Bommaraju, Kavanaugh, Hou and Bessett assert that abortion is often more difficult to access than other types of reproductive health services in the US as a result of the convergence of “three major mutually-reinforcing factors: lack of public financing for abortion services, legislative efforts to restrict access, and stigma associated with the procedure” ([44] p. 62). Women who are members of “vulnerable” populations, including racial or ethnic minorities, youth, socioeconomically disadvantaged, underinsured, or those with certain medical conditions are known to be at greater risk of disparate health care access [45], and may have particular difficulty accessing abortion services. Such vulnerabilities have been associated with delays in care, and in some cases, the continuation of unwanted pregnancies [46, 47].

Rural-residing women generally have less access to health care compared to those who live in urban areas [33, 38], and this pattern is also found with abortion services. In 2014, 90% of US counties - where 39% of all US women lived – had no abortion provider [47] legislation specifically aimed at regulating abortion providers (referred to as Targeted Regulation of Abortion Providers or “TRAP” laws) has led some to stop providing services altogether [47, 48] resulting in increases in average travel distances for women in some states [48]. Overall, TRAP laws have been associated with delays in obtaining or forgoing abortion care altogether, as well as increasing attempts at self-abortion by women [47–49]. Decreased access to abortion limits women’s ability to make the best decisions about childbearing for themselves and their families.

Women of lower socioeconomic status and women of color in the United States have higher rates of abortion than women of higher socioeconomic status and White women. These disparities are related to systemic hardships experienced by disadvantaged communities, including decreased access to health care, higher levels of stress, exposure to racial discrimination, and poorer living and working conditions [50, 51]. Disparities in abortion rates are related to disparities in unintended pregnancy, and associated disparities in contraceptive use. Structural factors, including economic disadvantage, neighborhood characteristics, lack of access to family planning services, and mistrust in the medical system underlay these disparities in abortion. Reduced access to abortion will result in more women experiencing later abortions or having an unintended childbirth which will worsen health disparities [52].

### Are abortions safe?

Overall, abortion is very safe; a first-trimester abortion is one of the safest medical procedures and carries minimal risk—less than 0.05%—of major complications that might need hospital care [53–55]. Mortality is extremely rare when abortion is performed by qualified, competent licensed healthcare providers and occurs early in pregnancy [56]. The risk of death associated with abortion increases with gestational age, from 0.3 per 100,000 abortions at or before eight weeks to 6.7 per 100,000 at 18 weeks or greater [56]. These rare deaths are usually the result of such things as adverse reactions to anesthesia, embolism, infection, or uncontrollable bleeding. In comparison, a woman’s risk of death during pregnancy and childbirth is ten times greater [56]. The abortion mortality rate was at least as low as the mortality rate associated with plastic surgery at licensed or accredited ambulatory surgical centers in the same decade, approximately equivalent to the proportion of marathon runners who died during races in the same time period [52, 57].

When compared against the risk of morbidity and mortality that occur during pregnancy for women over 35 years of age, abortion is a far safer option. Morbidity and adverse events during hospital delivery and post-partum maternal hospitalization increased 75% and 114%, respectively from 1998 to 1999 to 2008–2009 [58]. The risk of morbidity increases with maternal age, ranging, for example, from 6.6% for preeclampsia to 18.6% for obesity [59]. The risk for negative infant outcomes, such as stillbirth, disability, and prenatal demise, increases as well [59]. The most common types of morbidity associated with pregnancy for midlife women are hypertensive disorders of pregnancy [59]. Among almost 55,000 women, morbidity identified across service settings were experienced by 2.1% who had a medication abortion, and by 1.3% in the first trimester and 1.5% in the second trimester or later among those who had an abortion procedure [34].

Serious abortion related adverse events (clinical errors) or morbidity (conditions due to pregnancy or abortion process) are rare and are defined as those resulting in intervention (surgical repair for uterine perforation, transfusion for hemorrhage) occur less than 0.1%) and hospitalization for pelvic infection/sepsis or hemorrhage occur less than 0.5%. [34]. The most common non-serious (minor) adverse event (incomplete abortion) or morbidity (continued uterine bleeding) often requires re-aspiration or repeat abortion in an outpatient setting [60]. Other potential adverse events or morbidity diagnoses following abortion or with midlife pregnancy are provided in Table 7.

**Table 7:** Adverse Events/Morbidities Associated with Abortion and Pregnancy

Adverse Events/ Associated with Abortion	* Maternal Morbidities Associated with Pregnancy in Women over 35 Years of Age
Anesthesia side-effects Incomplete abortion (retained products of conception) Continuing pregnancy/Missed Ectopic Pregnancy Pelvic Infection Hemorrhage (> 500 cm^3^ uterine bleeding) resulting from uterine perforation Uterine/cervical perforation Bleeding/Disseminated intravascular coagulation (DIC) Bowel or bladder injury Cervical shock	Acute Renal Failure Cesarean section delivery Gestational Diabetes/Diabetes Hemorrhage Hypertension disorders of pregnancy GU Infections Hematometra Hemorrhage (> 500 cm^3^ uterine bleeding) Myocardial Infarction Obesity and Weight Gain Placenta Previa Preeclampsia Preterm Labor Pulmonary Embolism Respiratory Distress Syndrome Shock

Callaghan, Creanga, & Kuklina, 2012; Franz & Husslein, 2010; Grossman, Anderson, et al., 2015; Hand, 2014; Lim & Singh, 2014; Raymond, Grimes, 2012; Raymond, Grossmam, & Weaver, 2014; Taylor, et al., 2017

*Maternal complications only

## Tertiary prevention of unintended pregnancy

### What is tertiary prevention of unintended pregnancy?

Tertiary prevention of unintended pregnancy emphasizes prevention of adverse events associated with a later term unintended pregnancy, supporting women and their families who experience later term unwanted pregnancy and may include prevention efforts for women who have had multiple unintended pregnancies. Women who are at high risk, including those who carry an unwanted pregnancy to term or experience a later term abortion will require more professional attention and care coordination than women having an unintended pregnancy that is diagnosed very early in gestation. Although public financing for prenatal care has been expanded, there continue to be documented disparities in receipt of unplanned pregnancy care and disparities in maternal and infant outcomes by race and SES [61]. Improving the accessibility and quality of unintended pregnancy care can further ensure that all women who continue their unplanned pregnancies have the best possible pregnancy and parenting outcomes. A tertiary approach to preventing unintended pregnancy should be combined with multifaceted public health interventions addressing health disparities in reproductive health services, toxic stress, and economic supports [13].

## What might the future hold for reproductive Health Research, rights, and justice?

### What are the future issues for research related to unintended pregnancy prevention and midlife women?

At this writing and without a crystal ball, we can only guess at the future of unintended pregnancy prevention and care as it relates to all women and specifically midlife women. What we do know is that in the current political climate there are likely to be fewer resources for research and increasing political interference and disparities with access to quality sexual and reproductive health care.

The consequences of political interference for women’s reproductive health and justice not only limit care options, but also impose restrictions on research [61]. Harris investigated the consequences of antiabortion politics trumping science by questioning the legitimacy of abortion research and stigmatizing the status of the work. Moreover, in addition to deterring investigators from studying abortion through limiting federal funding for the research, in vitro fertilization (IVF) research and research on human embryonic stem cell lines has also been limited by policies affecting what the National Institutes of Health is empowered to fund. Consequences of these policies has been limitation of access for women and families to knowledge that would inform a wide range of reproductive health issues spanning a range from establishing a pregnancy to ending one [39, 61]. Harris has pointed out the threat to reproductive justice that has been imposed by limiting research on topics such as IVF to private funding sources and the consequent access to IVF to a subset of women with financial resources to obtain IVF services and thus contribute to IVF research efforts. Balancing research needs for contraception, all unintended pregnancy prevention interventions, and fertility enhancement during midlife in federal research portfolios would serve the goal of reproductive justice.

At this time, we have identified many important questions for research pertaining to midlife women and unplanned pregnancy. Contemporary research on the menopausal transition and early postmenopausal has provided novel research findings, positioning scientists to understand more fully the optimal approaches to prevention of unplanned pregnancy for midlife women. Appreciation of the influence of chronic illness, in particular multiple chronic illnesses on midlife women’s health could contribute to refinement of prevention strategies to help women avoid unintended pregnancy while minimizing their risk of adverse outcomes related to the use of some types of contraceptives or decisions to have sterilization procedures. At the same time, expanded research about contemporary midlife women’s sexual behavior patterns could enrich the evidence on which primary prevention of unplanned pregnancy is based, by providing data about US women comparable to that available in Europe. In addition, understanding the consequences for midlife women of carrying an unintended pregnancy to term and parenting an infant would benefit from additional research on which to base approaches to care, such as tertiary prevention of unintended pregnancy. In particular, advancing understanding of repeated unintended pregnancy and factors that interfere with women’s ability to manage their fertility warrant attention in national data gathering efforts that would drive research agendas [3]. Finally, recent research findings from an evaluation of abortion services point to the need for further research to improve services offered to women. Pain management challenges for over 5000 women having early aspiration abortions was a common theme in women’s descriptions of their experiences, as was the need to address the stigma and shame many of these women felt about their need for abortion [62].

What concerns are there about Reproductive Health? Rights, Justice, and Politics.

Republican majorities in the federal government and in most states are putting at risk existing protections for abortion, parenting, and birth control rights. Midlife women have an opportunity to help shape policy that affects not only their portion of the population, but all women.

Before abortion became legal through the Supreme Court case Roe v. Wade, an estimated 1.2 million women per year sought illegal abortions. Today, while legal abortion is safe, there has been increasing political interference (legislation, regulations) that threatens reproductive health access. Within the past decade alone, US women have experienced both increased and reduced options related to sexual and reproductive health care. With the passage of the Affordable Care Act in 2010, women were assured that contraceptive methods would be available to them as an insurance benefit without co-payment and that insurers would be obligated to provide coverage/ benefits to women instead of viewing them as having a pre-existing condition – being a woman [5]. With the weakening of policy insuring contraceptive access as a result of a Supreme Court Case (Burwell vs Hobby Lobby) which allowed employers to limit contraceptive benefits in health insurance based on the employers’ religious beliefs, women’s access to contraceptives as envisioned in the original ACA was limited. In addition, increasing restrictions on access to abortion in state legislation and regulation, such as those limiting the types of facilities in which abortions could be performed, further threatens women’s reproductive choice. Indeed, in some states, politicians have pushed for laws and regulations restricting ethical standards of care for women and are imposing politics and ideology on evidence-based clinical care as outlined by the recent policy report “Politics in the Exam Room” led by a coalition of 24 nursing, medical, health and advocacy organizations, the Coalition to Protect the Patient-Provider Relationship (http://www.coalitiontoprotect.org). For more detailed information about policy resources related to reproductive health, see Table 8.

**Table 8:** Reproductive Health Policy Resources

Resource	Location of Resource
Reproductive Health Policy Resources	http://www.womenshealthpolicyreport.org/articles/daily.html?referrer=http://go.nationalpartnership.org/site/PageServer?pagename=report_daily http://www.nationalpartnership.org/our-impact/
Policy advocacy/action	http://www.reprohealthwatch.org/?referrer=http://www.womenshealthpolicyreport.org/articles/monthly/#5
Contraception policy	http://www.nationalpartnership.org/issues/repro/birth-control.html
Abortion policy	http://www.nationalpartnership.org/issues/repro/abortion.html
Impact of abortion restrictions	http://www.scholarsstrategynetwork.org/scholar-spotlight/what-trump-means-abortion-access
What if Roe fell? Impact at state level (Center for Reproductive Rights)	https://www.reproductiverights.org/sites/default/files/documents/Roe_PublicationPF4a.pdf
Organizations working toward reproductive justice like the	National Network of Abortion Funds, ACCESS Reproductive Justice, Sister Song, and Sister Reach.

In addition, politics has trumped evidence supporting who may provide abortion services [63]. A strategy to reduce access to abortion has been state regulation limiting the type of health care provider who may provide medication and aspiration abortion. A recent study evaluating the outcomes of over 11,000 early aspiration abortions completed by physicians, and newly trained nurse practitioner, certified nurse midwives, and physician assistants in California revealed that abortion adverse effects were clinically equivalent among these groups of health professionals, supporting policies to allow these providers to perform early aspirations [60]. Moreover, updated findings from this study based on over 16,000 women having early aspiration underscore the very low rate of adverse complications/effects associated with this procedure [64].. Changing state regulations to improve access to early aspiration abortion for women by expanding the types of health professionals allowed to provide the service would seem a logical next step.

As a consequence of increasing threat to women’s reproductive health rights, future options for midlife women’s management of unintended pregnancy may be constrained. With political threats to defund Planned Parenthood, one of the primary resources for women’s reproductive health care is at risk and with it a resource providing US women with the full range of approaches to preventing unintended pregnancy. Protecting reproductive rights and promoting reproductive justice for women demands activism at all levels of the society. The political becomes personal as midlife and younger women may find themselves unable to access a broad range of unintended pregnancy prevention services. Limitations on women’s access to these services intersect with social determinants of their health, ultimately affecting women who are most vulnerable and who already suffer from marginalization and discrimination in the larger society. Using strategies to socially construct fertile women as “welfare queens” and “teen moms” has reinforced the political disenfranchisement of the population of all fertile women, contributing to our national failure to create effective reproductive health policy [65].

There is an urgent need to expand and protect policies that insure access to care to help women prevent unintended pregnancy. Moreover, there is an urgent need to protect the patient-provider relationship, insuring that health care professionals are not limited in providing the care to midlife women that they need and desire. Research and policy that recognizes the importance of all aspects of women’s reproductive health—including pregnancy prevention, abortion care, pregnancy services, and economic supports—are essential to meeting the sexual and reproductive health care needs of low-SES women and women of color [39].

In the current political maelstrom, US women will continue to experience unintended pregnancies, in some parts of the country without access to the option of pregnancy termination. In addition, many women who have benefitted from the access to coverage of contraception by health insurance policy changes provided by the Affordable Care Act may find themselves with more limited access, if not inability to obtain affordable contraception. Likely consequences are that unintended pregnancy will continue to be a problem for many women in one of the world’s most developed nations. In addition, public and private insurance coverage for abortions especially for low income women and women of color remains in jeopardy depending on contemporary political targets.
